# A Comprehensive Infrastructure for Big Data in Cancer Research: Accelerating Cancer Research and Precision Medicine

**DOI:** 10.3389/fcell.2017.00083

**Published:** 2017-09-21

**Authors:** Izumi V. Hinkson, Tanja M. Davidsen, Juli D. Klemm, Ishwar Chandramouliswaran, Anthony R. Kerlavage, Warren A. Kibbe

**Affiliations:** ^1^Center for Biomedical Informatics and Information Technology, National Cancer Institute Rockville, MD, United States; ^2^Science and Technology Policy Fellowship Program, American Association for the Advancement of Science Washington, DC, United States; ^3^Office of Genomics and Advanced Technologies, National Institute of Allergy and Infectious Diseases Bethesda, MD, United States; ^4^Department of Biostatistics and Bioinformatics, Duke University School of Medicine Durham, NC, United States

**Keywords:** genomics, proteomics, imaging, big data, cancer, precision medicine, cloud infrastructure

## Abstract

Advancements in next-generation sequencing and other -omics technologies are accelerating the detailed molecular characterization of individual patient tumors, and driving the evolution of precision medicine. Cancer is no longer considered a single disease, but rather, a diverse array of diseases wherein each patient has a unique collection of germline variants and somatic mutations. Molecular profiling of patient-derived samples has led to a data explosion that could help us understand the contributions of environment and germline to risk, therapeutic response, and outcome. To maximize the value of these data, an interdisciplinary approach is paramount. The National Cancer Institute (NCI) has initiated multiple projects to characterize tumor samples using multi-omic approaches. These projects harness the expertise of clinicians, biologists, computer scientists, and software engineers to investigate cancer biology and therapeutic response in multidisciplinary teams. Petabytes of cancer genomic, transcriptomic, epigenomic, proteomic, and imaging data have been generated by these projects. To address the data analysis challenges associated with these large datasets, the NCI has sponsored the development of the Genomic Data Commons (GDC) and three Cloud Resources. The GDC ensures data and metadata quality, ingests and harmonizes genomic data, and securely redistributes the data. During its pilot phase, the Cloud Resources tested multiple cloud-based approaches for enhancing data access, collaboration, computational scalability, resource democratization, and reproducibility. These NCI-led efforts are continuously being refined to better support open data practices and precision oncology, and to serve as building blocks of the NCI Cancer Research Data Commons.

## Introduction

Precision medicine has evolved out of the seminal work of the Human Genome Project, advancements in DNA sequencing technology, developments in high throughput and large-scale molecular biology technologies, improvements in the speed and scale of computation, and innovations in biomedical informatics. This progress has resulted in the molecular characterization of individual patient tumors, the identification of actionable genetic alterations, and the development of evidence-based molecular cancer diagnostics and targeted therapies. Although, cancer types have been traditionally classified by organ or cell type, with the aid of genomics, cancer patients are increasingly being treated according to their cancer's unique molecular signature. Cancer is a diverse array of genetically-driven diseases. The identification and validation of actionable genetic alterations including amplifications, rearrangements, and gain-of-function mutations, has spurred the use of genomic data in oncology practices. Targeted gene sequencing panels, for example, offer insight into the genetic drivers of an individual's tumor and inform the diagnosis, prognosis, and targeted treatment of cancer patients. A number of targets for drug development have been outlined previously (Hyman et al., 2017). Imantinib—a BCR-ABL inhibitor for chronic myelogenous leukemia, trastuzumab—a monoclonal antibody-based treatment for HER-2 positive breast cancer, vermurafenib—a mutated BRAF V600E inhibitor for metastatic melanoma, and many others serve as precision oncology success stories. Other candidate genes are currently under pre-clinical and clinical investigation for the development of targeted cancer therapies. Increasing our understanding of how molecular signatures are associated with treatment outcomes in patient populations, and translating these discoveries into the clinic, will improve treatment decisions for the individual.

In support of NCI's Precision Medicine in Oncology Initiative and the Beau Biden Cancer Moonshot, NCI is leading numerous multi-disciplinary efforts to accelerate the development of precision oncology diagnostics and treatments. Here, we describe a subset of ongoing NCI programs that combine biomedical big data, biotechnology, informatics, clinical research, and computer science to create new ways to more precisely study, predict, diagnose, and treat cancers.

## NCI programs provide big data resources to serve the cancer research community

The goal of precision oncology is to use each patient's unique collection of germline variants and somatic mutations to inform their diagnosis, prognosis, and therapy; working toward this goal, there has been a push toward large-scale, high throughput studies of patient-derived biospecimens.

Molecular profiling of patient-derived samples, including whole genome sequencing, has led to a data explosion that is contributing to our increased understanding of cancer driver genes, cancer molecular subtyping, cancer risk, therapeutic response, and treatment outcomes. NCI-supported programs such as The Cancer Genome Atlas (TCGA), Therapeutically Applicable Research to Generate Effective Treatments (TARGET), and Clinical Proteomic Tumor Analysis Consortium (CPTAC) have generated large datasets amassing petabytes of data. These, along with other datasets and resources described in this paper are available to researchers both in the US and internationally (Table 1).

**Table 1:** Selected NCI-supported projects.

**Project name**	**Lead institution(s)**	**Project URL**
The Cancer Genome Atlas (TCGA)	National Cancer Institute National Human Genome Research Institute	cancergenome.nih.gov
Therapeutically Applicable Research to Generate Effective Treatments (TARGET)	NCI Office of Cancer Genomics	ocg.cancer.gov/programs/target
Clinical Proteomic Tumor Analysis Consortium (CPTAC)	NCI Office of Cancer Clinical Proteomics Research	proteomics.cancer.gov/programs/cptac
Applied Proteogenomics Organizational Learning and Outcomes (APOLLO) Network	Department of Defense Department of Veterans Affairs National Cancer Institute	proteomics.cancer.gov/programs/apollo-network
The Cancer Imaging Archive (TCIA)	University of Arkansas for Medical Sciences NCI Division of Cancer Treatment and Diagnosis	www.cancerimagingarchive.net
Genomic Data Commons (GDC)	NCI Center for Cancer Genomics	gdc.cancer.gov
Database of Genotypes and Phenotypes (dbGaP)	National Center for Biotechnology Information	www.ncbi.nlm.nih.gov/gap
NCI Cloud Resources	National Cancer Institute	cbiit.cancer.gov/cloudresources
Broad FireCloud	Broad Institute	firecloud.org
Institute for Systems Biology Cancer Genomics Cloud (ISB-CGC)	Institute for Systems Biology	isb-cgc.org
Seven Bridges Cancer Genomics Cloud (SB-CGC)	Seven Bridges	www.cancergenomicscloud.org
NCI Cancer Research Data Commons	National Cancer Institute	cbiit.cancer.gov/cancerdatacommons

*NCI-supported projects annotated with lead institutions and URLs*.

In December 2005, TCGA was announced as a new collaboration between the NCI and the National Human Genome Research Institute (NIH, 2005). Building upon the pioneering work of the Human Genome Project, the two institutes embarked on a mission to explore the genomic changes that occur in human cancers. The overarching goal of TCGA was to increase our understanding of different cancer types to improve screening and treatments, and to build on this data to create new prevention strategies. TCGA includes the genomic analysis of 33 different tumor types and matched normal tissue from over 11,000 patients and has resulted in thousands of publications (Cancer Genome Atlas Research Network, 2008, 2011, 2012, 2017; Cancer Genome Atlas, 2012). Data types collected include DNA copy number arrays, DNA methylation, exome, and whole genome sequencing, mRNA arrays, microRNA sequencing, and reverse phase protein arrays, totaling ~2.5 petabytes of data.

TARGET was launched in 2006. TARGET's goal is to characterize the genome and transcriptome of hundreds of pediatric acute lymphoblastic leukemia, acute myeloid leukemia, Wilms tumor, clear cell sarcoma of the kidney, rhabdoid tumor, neuroblastoma, and osteosarcoma samples. Through genomic and transcriptomic analyses, researchers are studying the relationships among alterations at the DNA and RNA levels, cancer growth, cancer progression, and pediatric patient survival (Mullighan et al., 2009; Pugh et al., 2013; Eleveld et al., 2015). The TARGET project has performed whole genome sequencing on most samples collected and the entire dataset is in the petabytes range.

NCI's CPTAC aims to interrogate cancers at the protein level to link genotype to proteotype, with the goal of understanding the basis of cancer phenotypes. CPTAC's objectives are four-fold: (1) characterize the proteomes of tumor and normal tissues; (2) perform proteogenomic analyses of cancer biospecimens; (3) identify potential biomarker candidates through discovery proteomics and develop targeted assays against those candidates; and (4) perform verification tests on those targeted assays. Phase I of CPTAC consisted of technical quality assurance studies (Paulovich et al., 2010). Complementary to TCGA studies, CPTAC Phase II consisted of mass spectrometry-based proteomic analyses of TCGA breast, ovarian, and colorectal samples (Zhang et al., 2014, 2016; Mertins et al., 2016). The recently launched CPTAC Phase III is a proteogenomic analysis of prospectively collected tissues from additional cancer types. Furthermore, to support precision oncology, CPTAC Phase III has established Proteogenomic Translational Research Centers that will study the efficacy of cancer therapies on individual tumor samples to generate predictive models. CPTAC data currently totals ~16 TB of data, and upon completion of CPTAC III, this number is expected to increase four-fold to ~66 TB of data.

With the announcement of the Beau Biden Cancer Moonshot, the Applied Proteogenomics Organizational Learning and Outcomes (APOLLO) Network has emerged as a tri-agency collaboration to enable oncologists to use their patients' unique proteogenomic profiles to inform precision oncology treatments (Moonshot, 2016; OCCPR, 2016). Together with the Department of Veterans Affairs (VA) and the Department of Defense (DoD), NCI aims to perform proteogenomic analyses of a cohort of 8,000 cancer patients within the VA and DoD healthcare systems. These analyses will provide insight into the mutations and pathways that drive cancer progression and support the development of targeted and combination therapies. In the next 5 years, APOLLO is expected to amass petabytes of genomic, proteomic, imaging, and clinical data.

As the -omics sciences increase the volume of data collection, the need for big data solutions intensifies. To address this need, biomedical research has been moving toward data curation and data sharing models established by other big data fields such as astrophysics. Through major technological advancements, the Hubble Deep Field image marked a turning point in astrophysics where researchers led a concerted effort in data quality assessment, annotation, and curation. This work led to the development open source data resources, and user interfaces that obviated the resource intensive download of large datasets (Andersen, 2012). Biomedical informatics has reached a similar a turning point where key innovations in data storage and distribution such as compression algorithms, indexing systems, and cloud platforms must be leveraged.

## NCI genomic data commons and cloud resources

In addition to the data curation and storage needs of modern biomedical research, other challenges include the development of robust analytical tools, as well as infrastructure and funding models to support these efforts. As data generation expands, local storage, and computational solutions become less feasible. Thus, NCI has set out to build the NCI Cancer Research Data Commons (NCRDC), a cloud-based infrastructure in support of data sharing, tool development, and compute capacity to democratize big data analysis and to increase collaboration among researchers. NCI has sponsored two recent initiatives that serve as the foundation for the Cancer Research Data Commons—the Genomics Data Commons (GDC), and three Cloud Resources.

The GDC, built and managed by the University of Chicago Center for Data Intensive Science, in collaboration with Ontario Institute for Cancer Research, all under an NCI contract with Leidos Biomedical Research, is a unified genomic data repository that hosts authoritative NCI reference datasets such as TCGA and TARGET (Grossman et al., 2016; NIH, 2016). The primary goals of the GDC are to ensure data and metadata quality, ingest and harmonize genomic data, support data dissemination practices in alignment with Findable Accessible Interoperable Reusable (FAIR) principles (Mons et al., 2017), and securely redistribute data to researchers. In addition, the GDC takes part in collaborative efforts such as the Global Alliance for Genomics and Health (Knoppers, 2014). Through the GDC, researchers can download harmonized genomic data for analysis on their local servers. To bolster data sharing practices and streamline genomic data analysis, much of the genomic data stored at the GDC have been made available through the NCI Cloud Resources.

The NCI Cloud Resources were initially launched in 2016 as the Cancer Genomics Cloud (CGC) Pilots. The purpose of the CGC Pilots was to explore multiple cloud-based approaches for enhancing secure data access, collaboration, computational scalability, resource democratization, and reproducibility. Through this program, the Broad Institute, the Institute for Systems Biology, and Seven Bridges have each developed what are now known as Cloud Resources. Each platform is deployed in a commercial cloud, and has applied a distinct approach to providing access to TCGA and TARGET genomic data in a cloud environment, and integrating proteomic data from CPTAC as well as radiology images and associated metadata from The Cancer Imaging Archive (TCIA). In addition to providing access to these datasets through rich Application Programming Interfaces (APIs) and graphical user interfaces, the Cloud Resources each provide a platform to enable the deployment of analysis, visualization, and other computational tools in the cloud, bypassing the need to bring data to a local infrastructure. The Cloud Resources support tool deployment through the use of Docker containers, which allow users to package their tools along with all associated dependencies. These “containerized” tools can be connected and executed as workflows in these cloud environments. End user documentation provides users with guidance on how to query data, install tools, as well as create and run workflows in each environment. All three platforms conform to strict federal information system security requirements and manage access to controlled data through Database of Genotype and Phenotype (dbGaP) authorization. In addition to their fundamental charter of providing secure cancer genomic data access co-localized with analysis pipelines and visualization tools, the Cloud Resources each offer unique capabilities suitable for a range of research needs.

### Broad firecloud

The Broad Institute's FireCloud, was built as the next generation of Broad Institute's Firehose data analysis infrastructure developed for the TCGA program (Ulrich, 2016). FireCloud harnesses the elastic compute capacity of Google Cloud Platform for large-scale genomic analyses akin to those available through Firehose. Key advantages offered by FireCloud include running Broad's best practice tools and pipelines such as ContEst, MuTect, and Oncotator. FireCloud users can also access curated open and controlled-access TCGA workspaces, upload their own data, and share workspaces with collaborators. FireCloud also allows users to leverage the rich query interface of the GDC to create cohorts of interest and download data “just-in-time” to a FireCloud-based workspace for follow on analyses. Similar approaches are under development to support the analysis of CPTAC data and TCIA images. Researchers at the Broad Institute, in collaboration with IBM Watson, are using FireCloud to tackle one of precision oncology's toughest questions—which genomic signatures are linked to drug-resistant cancers (Park, 2016)? While targeted therapies are currently being applied in the clinic, oncologists have been unable to predict when a patient will no longer respond to a given line of therapy. The data analysis infrastructure provided by FireCloud directly supports researchers investigating problems such as this one to increase the efficacy of precision medicine for cancer patients.

### ISB-CGC

The Institute for Systems Biology Cancer Genomics Cloud (ISB-CGC) runs on the Google Cloud Platform and offers an interactive web-based application and hosts Application Programming Interfaces (APIs) such as the Global Alliance for Global Health API. ISB-CGC takes advantage of Google Cloud Platform's built-in resources such as BigQuery, Compute Engine, App Engine, Cloud Datalab, and Google Genomics. Researchers can use BigQuery to explore clinical, biospecimen, level-3 open access TCGA, and CPTAC II data. ISB-CGC hosts numerous genomics tools and has recently added the Trans Proteomic Pipeline analysis suite. Researchers can now access complementary genomic and proteomic data, run multi-omic analyses, and perform BigQuery searches to investigate genetic alterations, copy number, transcript expression, protein expression, and molecular pathways that are involved in cancer biology. ISB-CGC has also made radiology and tissue images from TCIA and the GDC available through Google Cloud Storage. Additional datasets available at ISB-CGC include the Catalog of Somatic Mutations in Cancer^1^ and the Cancer Cell Line Encyclopedia (CCLE)^2^. A recent publication in Nature Scientific Reports showcased a project which used the ISB-CGC to perform fast, cheap, and robust RNA-sequencing analyses of 12,307 samples from CCLE and TCGA (Tatlow and Piccolo, 2016). Authors, P. J. Tatlow and Dr. Stephen Piccolo, used preemptible virtual machines to analyze over 64 terabytes of TCGA data for only $0.09 per sample. The scalable, cost effective compute capabilities of ISB-CGC have enabled researchers to perform robust analyses of big data that will ultimately lead to the enhanced understanding of individual cancers.

### SB-CGC

Currently, over 1,600 researches from over 40 countries are using the Seven Bridges Cancer Genomics Cloud (SB-CGC) to analyze hosted genomic data, and/or their own data^3^. Dr. Julia Salzman's lab at Stanford University has deployed Mismatched Alignment CHimEra Tracking Engine (MACHETE) (Hsieh et al., 2017), a statistical algorithm for the detection of gene fusions, on the SB-CGC (Salzman, 2017). Using RNA-seq data from hundreds of TCGA samples, MACHETE was used to perform statistical modeling of fusion artifacts to precisely detect novel gene fusions including rare potential drivers of cancer. This research, fueled by cloud computing, is enabling precision oncology through the discovery of novel, potentially druggable gene fusions. In addition to the TCGA and TARGET data, SB-CGC hosts TCGA radiology images, CCLE data, as well as Simons Genome Diversity Project data^4^. Leveraging its Cancer Genomic Cloud work, Seven Bridges has partnered with the Blood Profiling Atlas in Cancer Consortium^5^, to develop the Blood Profiling Atlas Analysis Cloud and provide the research community with analysis algorithms for liquid biopsy.

### CGC pilot beta-testing and evaluation

The initial versions of The NCI Cloud Resources, the CGC Pilots, were created to maximize the value of cancer -omics data through harnessing multi-disciplinary expertise. Synergizing technologies from the fields of medicine, molecular biology, informatics, and cloud computing, the CGC Pilots have begun to transform how cancer data analysis is conducted. Researchers, both in the US and internationally, have been able to advantage of these cloud-based resources. To ensure the success of this project and to identify areas for improvement, the CGC Pilot and NCI teams established mechanisms to support early adopters' use of these platforms and to collect their feedback.

The CGC Pilots teams provided technical support to new users who sought to implement new tools, access data, or create collaborative workspaces. Through the three CGC Pilots, NCI provided cloud compute and storage “credits” to offset the costs of evaluation of these platforms by cancer researchers. These funds directly impacted the work of researchers such as post-doctoral scholar Dr. Brittany Lasseigne, at the HudsonAlpha Institute for Biotechnology in Huntsville, AL. Dr. Lasseigne used the SB-CGC, to study dosage effects, context dependency, and tissue specificity of tumor suppressors across human cancers in TCGA. These Cloud credits supported the use of the large-scale genomic datasets co-located with computational resources and analysis tools, and increased research efficiency for many early stage researchers like Dr. Lasseigne (Lasseigne, Personal Communication).

To evaluate the CGC Pilots and support on-going NCI-funded cancer research, NCI funded administrative supplements to the active grants of investigators performing genomics-based research. The Funding Opportunity Announcement, Supplements to Support Evaluation of the NCI Cancer Genomics Cloud Pilots (PA-15-305), funded projects to use one or more of the NCI CGC Pilots for ongoing research activities. Funds were awarded to investigators whose projects aimed to install and test the performance of new analysis tools on a CGC Pilot, upload locally-generated genomic data and perform analyses on a CGC Pilot, and/or perform analyses of hosted TCGA data. The researchers reported having a generally positive experience working on the CGC Pilots; however, as expected, some encountered technical hurdles. When those technical issues arose, the vast majority of the groups were able to resolve their problems by working directly with the CGC Pilot support teams. Each research group provided extensive feedback to the CGC Pilots and NCI teams on what elements of the CGC Pilots could be improved. The majority of the administrative supplement awardees reported that they plan to continue to use the CGC Pilots to accelerate their research and that the CGC Pilots have the potential to be a vital resource for the cancer research community. The activities and outcomes of projects funded through these supplements have helped inform NCI's decision to continue supporting this project beyond the pilot phase and to develop a more comprehensive computational infrastructure for -omics and other big data types.

### Future vision: NCI cancer research data commons

Cancer research in the era of big data presents major challenges: computing on large datasets, combining expertise from various disciplines, and developing the infrastructure needed to enhance research efficiency. Recognizing the importance and urgency of these needs, the Beau Biden Cancer Moonshot Blue Ribbon Panel has recommended that the cancer research community aim to, “collect, share, and interconnect a broad array of large datasets so that researchers, clinicians, and patients will be able to both contribute and analyze data, facilitating discovery that will ultimately improve patient care and outcomes (BRP, 2016).” In line with this recommendation, the NCI is taking steps toward establishing the NCRDC, with the GDC and the Cloud Resources serving as the foundation for this vision.

**Figure 1:**
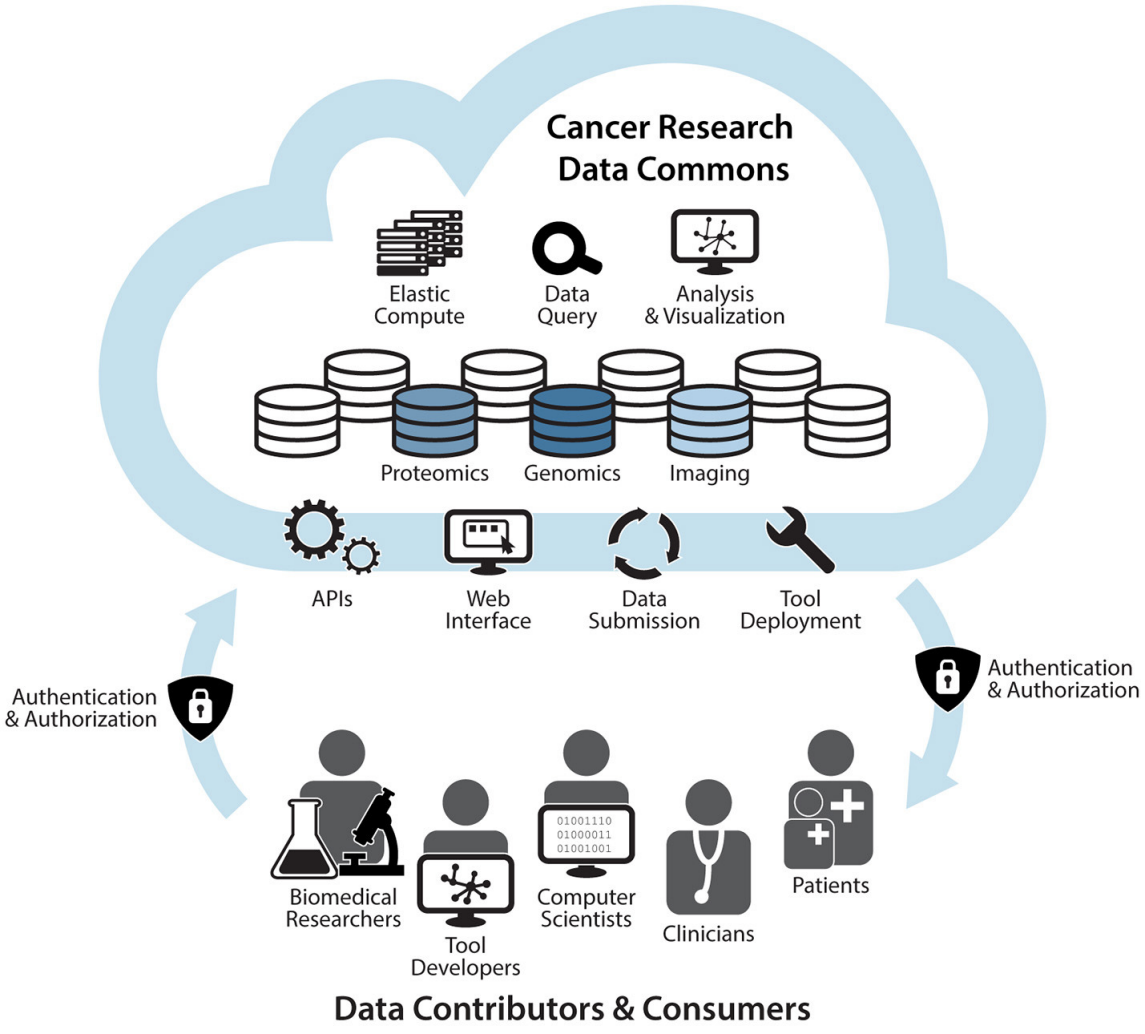
The NCI Cancer Research Data Commons: An Expandable Infrastructure. The NCI Cancer Research Data Commons will be a cloud-based network in which each node is focused on a specific data type. Nodes will include the Genomic Data Commons, Proteomic Data Commons, and Imaging Data Commons. Future plans include the addition of nodes that support other research modalities such as clinical data, epidemiological data, and cancer models. Through a secure authentication and authorization process, biomedical researchers, tool developers, computer scientists, informaticians, clinicians, and patients will be able to bring their own data and tools to nodes, as well as access harmonized data and hosted tools via APIs and a web interface. Users will also be able to harness elastic compute capabilities for computational analyses, visualization of results, and data queries in the cloud (NCI, 2017).

The GDC and Cloud Resources currently support basic and translational research, primarily using genomic and clinical data. These activities serve as the building blocks of the cloud-based NCRDC (Figure 1). The NCRDC will consist of multiple “nodes,” or digital knowledge bases with functionalities like those of the GDC and Cloud Resources. NCRDC nodes will each be centered on different research and clinical data types such as genomics, proteomics, imaging, cancer models, and epidemiology. Each node will house annotated datasets, raw data files, metadata, analysis, and visualization tools, as well as individual and collaborative workspaces. NCRDC users will be able to access authoritative datasets generated by NCI funded programs such as TCGA, TARGET, CPTAC, APOLLO, and TCIA. Each node will also employ a standardized process for data submission and quality control that will allow for the harmonization of new data, including user-generated data. Containerized tool deployment will also be supported by each Data Commons node. Each node will provide consistent, well-defined identifiers and semantics for access to data housed in that node and provide broadly-available computational support critical to the demands of modern cancer research and precision oncology. The Data Commons will thus support cancer research across multiple domains and platforms, allow for these data to be queried and analyzed in an integrated, secure, cross-domain manner, and provide the mechanisms for new data sources to be incorporated as they are generated. Through fostering community-driven, open-development informatics initiatives, the Cancer Research Data Commons will create, maintain, and extend informatics infrastructure and standards to improve connectivity among disparate information systems. Combining innovation, cloud computing, big data, and FAIR principles, this robust infrastructure will provide significant support for NCI's Precision Medicine in Oncology Initiative and the Beau Biden Cancer Moonshot by accelerating the discovery of novel therapeutic targets and disease biomarkers for individual cancer patients.

The era of big data in biomedical research and precision oncology calls for creative strategies borrowed from multiple scientific and technological disciplines. The GDC and Cloud Resources are important steps in supporting the next generation of data-driven cancer research. Looking ahead, the NCRDC represents an interdisciplinary solution to the challenges of big data in cancer research. NCI will continue to lead open science efforts toward the goals of improving prevention strategies, developing targeted diagnostics and therapeutics, and reducing the burden of cancer on patients, their families, and society.

## Author contributions

IH: Manuscript writing, figure design. TD, JK, AK, and WK: Manuscript and figure revision, approval of final manuscript.

### Conflict of interest statement

The authors declare that the research was conducted in the absence of any commercial or financial relationships that could be construed as a potential conflict of interest.

## Footnotes

^1^cancer.sanger.ac.uk/cosmic

^2^portals.broadinstitute.org/ccle

^3^http://www.cancergenomicscloud.org/usage

^4^docs.cancergenomicscloud.org/docs

^5^http://www.bloodpac.org
