# Corrigendum: A Comprehensive Infrastructure for Big Data in Cancer Research: Accelerating Cancer Research and Precision Medicine

**DOI:** 10.3389/fcell.2017.00108

**Published:** 2017-12-07

**Authors:** Izumi V. Hinkson, Tanja M. Davidsen, Juli D. Klemm, Ishwar Chandramouliswaran, Anthony R. Kerlavage, Warren A. Kibbe

**Affiliations:** ^1^Center for Biomedical Informatics and Information Technology, National Cancer Institute, Rockville, MD, United States; ^2^Science and Technology Policy Fellowship Program, American Association for the Advancement of Science, Washington, DC, United States; ^3^Office of Genomics and Advanced Technologies, National Institute of Allergy and Infectious Diseases, Bethesda, MD, United States; ^4^Department of Biostatistics and Bioinformatics, Duke University School of Medicine, Durham, NC, United States

**Keywords:** genomics, proteomics, imaging, big data, cancer, precision medicine, cloud infrastructure

Ishwar Chandramouliswaran (currently National Institute of Allergy and Infectious Diseases) was not included as an author in the published article. The authors apologize for this error and state that this does not change the content of the article.

The original article has been updated.

## Author contributions

IH: Manuscript writing, figure design. TD, JK, AK, and WK: Manuscript and figure revision, approval of final manuscript. IH, TD, JK, IC, AK, and WK: Management and project support for Cancer Genomics Cloud Pilots.

### Conflict of interest statement

The authors declare that the research was conducted in the absence of any commercial or financial relationships that could be construed as a potential conflict of interest.

